# Comparison of Manual and Semi-Automatic [^18^F]PSMA-1007 PET Based Contouring Techniques for Intraprostatic Tumor Delineation in Patients With Primary Prostate Cancer and Validation With Histopathology as Standard of Reference

**DOI:** 10.3389/fonc.2020.600690

**Published:** 2020-12-07

**Authors:** Simon K. B. Spohn, Maria Kramer, Selina Kiefer, Peter Bronsert, August Sigle, Wolfgang Schultze-Seemann, Cordula A. Jilg, Tanja Sprave, Lara Ceci, Thomas F. Fassbender, Nils H. Nicolay, Juri Ruf, Anca L. Grosu, Constantinos Zamboglou

**Affiliations:** ^1^ Department of Radiation Oncology, Medical Center–University of Freiburg, Faculty of Medicine, University of Freiburg, Freiburg, Germany; ^2^ German Cancer Consortium (DKTK), Partner Site Freiburg, Freiburg, Germany; ^3^ Institute for Surgical Pathology, Medical Center–University of Freiburg, Faculty of Medicine, University of Freiburg, Freiburg, Germany; ^4^ Department of Urology, Medical Center–University of Freiburg, Faculty of Medicine, University of Freiburg, Freiburg, Germany; ^5^ Department of Nuclear Medicine, Medical Center–University of Freiburg, Faculty of Medicine, University of Freiburg, Freiburg, Germany; ^6^ Berta-Ottenstein-Programme, Faculty of Medicine, University of Freiburg, Freiburg, Germany

**Keywords:** primary prostate cancer, focal therapy, contouring, PSMA-PET/CT, histopathology

## Abstract

**Purpose:**

Accurate contouring of intraprostatic gross tumor volume (GTV) is pivotal for successful delivery of focal therapies and for biopsy guidance in patients with primary prostate cancer (PCa). Contouring of GTVs, using 18-Fluor labeled tracer prostate specific membrane antigen positron emission tomography ([^18^F]PSMA-1007/PET) has not been examined yet.

**Patients and Methods:**

Ten Patients with primary PCa who underwent [^18^F]PSMA-1007 PET followed by radical prostatectomy were prospectively enrolled. Coregistered histopathological gross tumor volume (GTV-Histo) was used as standard of reference. PSMA-PET images were contoured on two ways: (1) manual contouring with PET scaling SUVmin-max: 0–10 was performed by three teams with different levels of experience. Team 1 repeated contouring at a different time point, resulting in n = 4 manual contours. (2) Semi-automatic contouring approaches using SUVmax thresholds of 20–50% were performed. Interobserver agreement was assessed for manual contouring by calculating the Dice Similarity Coefficient (DSC) and for all approaches sensitivity, specificity were calculated by dividing the prostate in each CT slice into four equal quadrants under consideration of histopathology as standard of reference.

**Results:**

Manual contouring yielded an excellent interobserver agreement with a median DSC of 0.90 (range 0.87–0.94). Volumes derived from scaling SUVmin-max 0–10 showed no statistically significant difference from GTV-Histo and high sensitivities (median 87%, range 84–90%) and specificities (median 96%, range 96–100%). GTVs using semi-automatic segmentation applying a threshold of 20–40% of SUVmax showed no significant difference in absolute volumes to GTV-Histo, GTV-SUV50% was significantly smaller. Best performing semi-automatic contour (GTV-SUV20%) achieved high sensitivity (median 93%) and specificity (median 96%). There was no statistically significant difference to SUVmin-max 0–10.

**Conclusion:**

Manual contouring with PET scaling SUVmin-max 0–10 and semi-automatic contouring applying a threshold of 20% of SUVmax achieved high sensitivities and very high specificities and are recommended for [^18^F]PSMA-1007 PET based focal therapy approaches. Providing high specificities, semi-automatic approaches applying thresholds of 30–40% of SUVmax are recommend for biopsy guidance.

## Introduction

Accurate intraprostatic tumor contouring is pivotal for successful delivery of high precision focal therapies of primary prostate cancer (PCa) and biopsy guidance. Radiation dose escalation has been shown to be beneficial for treatment outcome (1–3) and boosting visible tumor burden is currently being investigated in phase III trials (4, 5). Besides multiparametric magnetic resonance tomography (mpMRI) (6), positron emission tomography with tracers against prostate membrane specific membrane antigen (PSMA-PET) has emerged as an excellent technique for diagnostic and staging of primary and recurrent PCa (7–10). In primary PCa results from our workgroup as well as other studies suggest that PSMA-PET shows better sensitivities with comparable specificity than mpMRI in intraprostatic lesions detection (11, 12), gives complementary information (13) and may thus be favorable for focal therapy guidance (14). Different contouring approaches for Gallium-68-labeled ([^68^Ga]PSMA-PET) have already been validated and manual contouring applying SUVmin-max: 0–5 provided high sensitivities (11). Fluorine-18-labeled Tracers ([^18^F]PSMA-1007) have been implemented in nuclear medicine practice, with suspected benefits due to lesser renal elimination and consequent less background signal in the bladder (15) and performed with good diagnostic accuracy (16). Since [^68^Ga]- and [^18^F]PSMA-1007 tracers show differences in SUV distribution scaling recommendations might not be used interchangeable (17). This prospectively designed study aims to validate [^18^F]PSMA-1007 PET based contouring approaches for intraprostatic tumor contouring using whole mount histopathology as standard of reference, since a consensual method to accurately contour intraprostatic lesions for this tracer has not yet been established.

## Patients and Methods

### Patients

Between June 2019 and February 2020, 10 patients with histopathological proven primary PCa, pre-therapeutic [^18^F]PSMA-1007 PET scan and intended radical prostatectomy were prospectively enrolled. Exclusion criteria were neoadjuvant androgen deprivation therapy and transurethral prostate resection prior to PET imaging. See **Table 1** for patient characteristics. Written informed consent was obtained from all patients. The study was approved by the institutional review board (No. 476/19).

**Table 1:** Patient characteristics.

Patient	Age (y)	PSA (ng/ml)	pT	Gleason score (specimen)
1	70	4.3	pT2a	4+3 (7b)
2	66	17.2	pT3	4+3 (7b)
3	69	103.0	pT3a	4+5 (9)
4	76	5.0	pT2c	4+3 (7b)
5	80	8.6	pT2a	4+5 (9)
6	53	72.0	pT3b	5+4 (9)
7	64	19.5	pT3a	4+4 (8)
8	72	24.8	pT2a	4+4 (8)
9	74	13.9	pT3a	4+3 (7b)
10	66	17.5	pT3b	4+5 (9)
Median	70	17.4		
95% CI	64–74	5.3–51.9		

### PET Imaging

[18F]PSMA-1007 had been synthesized according to Cardinale et al. (18). The mean injected activity of [^18^F]PSMA-1007 was 299 MBq (min–max: 249–370 MBq). Patients underwent a whole-body PET scan starting 2 h after injection. Scans were performed with a 16-slice Gemini TF big bore in one patient and a Vereos PET/CT scanner in nine patients (all Philips Healthcare, USA). A phantom study was performed and comparable SUV values were obtained in both systems (19). Both scanners fulfilled the requirements indicated in the European Association of Nuclear Medicine (EANM) imaging guidelines and obtained EANM Research Ltd (EARL). accreditation during acquisition. At the time of the PET scan, either a contrast-enhanced or native diagnostic CT scan (120 kVp, 100–400 mAs, dose modulation) was performed for attenuation correction (depending on previous CT scans and contraindications). Please see (19) for details about reconstruction methods. All systems resulted in a PET image with a voxel size of 2 × 2 × 2 mm^3^. The uptake of [^18^F]PSMA-1007 was quantified in terms of standardized uptake values (SUV) normalized body weight.

### Histopathology and PET/CT Image Coregristation

PCa lesions in whole mount histopathology specimens were transferred into 3D volumes using a published in-house coregistration protocol and served as standard of reference (11, 20, 21). Following formalin fixation, the resected prostate underwent *ex-vivo* CT scan using a customized localizer. A customized cutting device was used to cut step sections every 4 mm to guarantee equal cutting angles between tissue specimen and *ex-vivo* CT-slices. After paraffin embedding specimens were cut using a Leica microtome. Hematoxylin and eosin staining was performed following routine protocols and PCa lesions were marked by one experienced pathologist. Histopathological slices were registered on *ex-vivo*-CT images and PCa contours were transferred into the corresponding CT images. Contours were interpolated by 2 mm expansion in Z-axis directions do create a model for 3D distribution. Manual coregistration allowing elastic deformations was used to take into account non-linear transformations of the prostatic gland after resection. Subsequently in this approach, following previously used workflows, manual coregistration of *ex-vivo* CT, including 3D volumes of pathology reference on *in-vivo* CT from diagnostic PSMA-PET/CT was performed. Pre-treatment mpMRIs (T2w sequences) were co-registered to *in-vivo* CT and an experienced radiation oncologist delineated prostatic gland on the CT-images under consideration of the mpMRI information.

### PET Based Contouring

**Figure 1:**
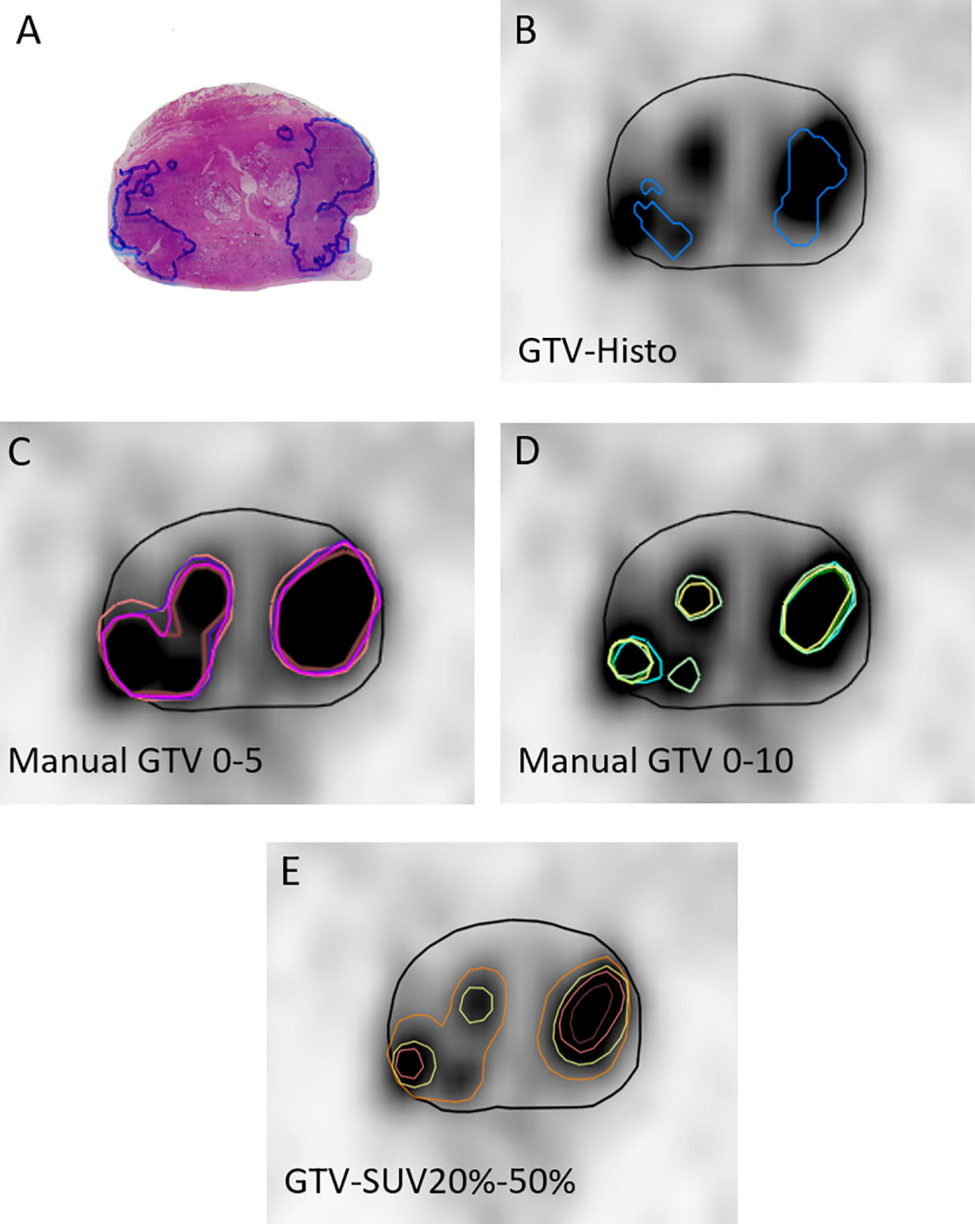
Image segmentations. **(A)** shows the H&E stained whole-mount prostatectomy specimen with intraprostatic tumor lesions marked in blue. The other images display representative axial PSMA-PET images with the respective GTVs. PET image scaling is SUVmin-max 0–5. **(B)** shows GTV-Histo. **(C)** shows manual contouring approaches with scaling SUVmin-max 0–5 (team 1 = brown, team 2 = pink, team 3 = magenta, and team 1v2 = purple). **(D)** shows manual contouring approaches with scaling SUVmin-max 0–10 (team 1 = cyan, team 2 = green, team 3 = yellow, and team 1v2 = dark green). **(E)** shows semi-automatic contouring approaches applying a threshold of SUVmax of 20% (orange), 30% (yellow), 40% (pink), and 50% (brown). Prostatic gland is marked in black.

Gross tumor volume (GTV) contouring of intraprostatic tumor lesions was performed in PSMA-PET images for all 10 patients using manual and semi-automatic approaches (**Figure 1**). Eclipse™ Treatment Planning System (Varian, USA) was used for both approaches. Three teams were recruited. Team 1 consisted of two readers with <1 year of experience in interpretation of PSMA-PET images. Team 2 consisted of two readers with >4 years of experience in interpretation of PSMA-PET images and team 3 consisted of one reader with <2 years of experience in interpretation of PSMA-PET images respectively. Additionally Team 1 repeated contouring blinded to previously performed segmentations after >4 months (Team 1v2).

Manual Contouring: According to recommendations for [^68^Ga]-PSMA-PET imaging scaling of SUVmin-max 0–5 was firstly applied for intraprostatic tumor lesion contouring (11). Due to volume overestimation and differences in SUVuptake between both tracers (17, 22), a second analysis applying individual scaling was performed to define an additional scaling range for manual contouring, which results in absolute volumes (GTV-Individual) more consistent with GTV-Histo. Therefore, PET images of each patient were scaled individually by modifying the scaling value of the upper SUV-limit, adjusting the volume to the available histological information. Based on the median applied SUVmax of 10, a second manual contouring approach with scaling SUVmin-max 0–10 was performed by all teams. Apart from PET and CT images no additional clinical information was provided.

Threshold Segmentations: A threshold of 20, 30, 40, and 50% of intraprostatic SUV max was applied for semi-automatic segmentation of GTV-20–50%, respectively.

All contours were created in the PET images and transferred to the corresponding, hardware-based, co-registered CT images. GTVs were trimmed to the prostatic gland and to the region of the prostatic gland, which was used for histopathologic examination.

### Statistical Analysis

Sensitivity and specificity for all GTVs based on the histology as reference were calculated by dividing the prostate in each CT slice into four equal quadrants as performed previously by our group (11). The statistical analysis was performed on GraphPad Prism v8.4.2 (GraphPad Software). Normal distribution was tested using the Shapiro-Wilk test. Since tested variables showed no Gaussian distribution Friedman test and uncorrected Dunn’s test at a significance level of 0.05 was used. Overlap of contours as well as the proportion of the GTVs to the prostatic gland was measured in the Eclipse planning software. Analyses of volumes including GTV-Histo was limited to the proportion of the prostate that was used for histopathological examination, defined by histological slices. Additionally, proportion of contoured GTVs and the whole prostate was calculated. Agreement between manual contours of team 1, team 2, team 3, and team 1v2 was assessed at voxel level using the Dice Similarity Coefficient (DSC), which is identical to the kappa index when applied at voxel level (23).

## Results

**Figure 2:**
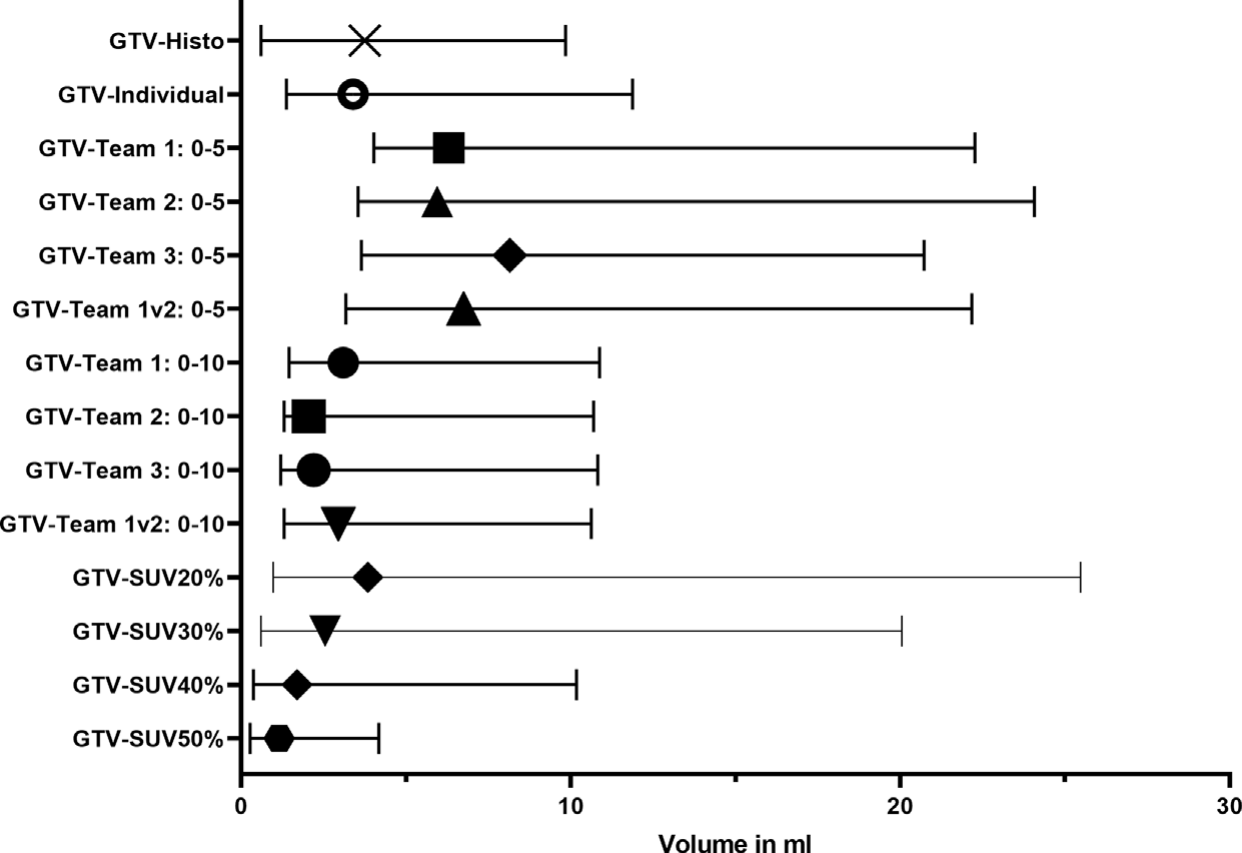
Volumes of histology reference (GTV-Histo) and different [^18^F]PSMA-1007 PET based segmentation approaches. The median and interquartile ranges over all patients are shown.

**Figure 3:**
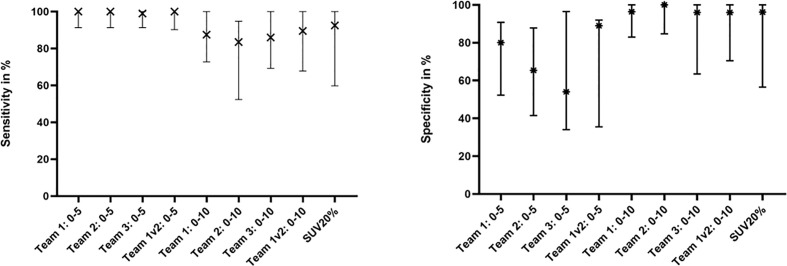
Sensitivity and Specificity of Team 1, Team 2, Team 3, Team 1v2, and SUV20%. The median and interquartile ranges over all patients are shown.

**Table 2** gives an overview of the absolute volumes, coverage of GTV-Histo, sensitivities and specificities of the evaluated contouring approaches. GTVs from scaling SUVmin-max 0–5 were significantly larger than GTV Histo (median 3.8 ml for GTV-Histo, median 6.2–8.2 ml for all teams, p = <0.0029, see **Figure 2**). Sensitivities were very high (median ≥99%) and specificities moderate (median 54–89%) (**Figure 3**). In the second step, individual PET image scaling for manual contouring revealed a varying SUVmax of 6–20 (corresponding to a percentage of SUVmax between 15 and 60%). Median applied SUVmax was 10 (corresponding to a median percentage of SUVmax of 36%). Median volume of GTV-Individual was 3.4 ml (**Figure 2**) and showed no statistically significant difference to GTV-Histo. Likewise median volumes of GTVs from scaling SUVmin-max 0–10 (median 2.6 ml, range 2.0–3.1 ml) were not statistically significant difference to GTV-Histo (**Figure 2**). Scaling SUVmin-max 0–10 and individual scaling achieved lower median sensitivities (84–90% for all teams, 89.0% for individual scaling, respectively) with higher median specificities (96–100% for all teams, 96% for individual scaling, respectively) (**Figure 3**). Sensitivities for scaling SUVmin-max 0–5 were mostly significantly higher than for scaling SUVmin-max 0–10 and individual scaling, *vice versa* specificities were mostly significantly lower (see **Table S1** for details about p = values). Analysis of the different manual segmentation methods revealed an excellent interobserver agreement with median DSCs between 0.87 and 0.94 (see **Table 3** for details). For proportion of prostate specimen, proportion of whole prostate and coverage of GTV-Histo please see **Table 2**.

**Table 2:** Overview of different [^18^F]PSMA-1007 PET based segmentation approaches in comparison with histology as reference standard.

	Median volume in ml (IQR)	GTV trimmed to specimen/prostatic specimen volume in % Median (IQR)	GTV/prostatic volume in % Median(IQR)	Coverage of GTV-Histo in % (IQR)	Median sensitivity in % (IQR)	Median specificity in % (IQR)
**GTV-Histo**	3.8 (0.6–9.9)	11 (3–29)				
**GTV-Team 1: 0–5**	6.3 (4.0–22.3)	29 (14–51)	29 (10–47)	72 (56–93)	100 (91–100)	80 (52–91)
**GTV-Team 2: 0–5**	6.2 (3.6–24.1)	30 (12–55)	31 (9–53)	72 (66–95)	100 (91–100)	65 (42–88)
**GTV-Team 3: 0–5**	8.2 (3.7–20.7)	34 (13–54)	29 (8–48)	75 (57–94)	99 (91–100)	54 (34–97)
**GTV-Team 1v2: 0–5**	6.7 (3.2–22.2)	32 (11–51)	30 (8–34)	78 (58–93)	100 (90–100)	89 (36–92)
**GTV-Team 1: 0–10**	3.1 (1.5–10.9)	11 (6–28)	12 (4–35)	59 (34–86)	88 (73–100)	96 (83–100)
**GTV-Team 2: 0–10**	2.1 (1.3–10.7)	10 (4–27)	8 (3-34)	56 (27–85)	84 (52–95)	100 (85–100)
**GTV-Team 3: 0–10**	2.2 (1.2–10.8)	11 (4–27)	8 (3–34)	53 (25–84)	86 (69–100)	96 (64–100)
**GTV-Team 1v2: 0–10**	3.0 (1.3–10.6)	34 (13–54)	9 (3–34)	59 (30–84)	90 (68–100)	96 (71–100)
**GTV-Individual**	3.4 (1.4–11.9)	13 (7–31)	10 (4–31)	56 (36–81)	89 (74–97)	96 (75–100)
**GTV-SUV20%**	3.9 (1.0–25.5)	19 (4–59)	21 (3–62)	69 (42–84)	93 (60–100)	96 (57–100)
**GTV-SUV30%**	2.6 (0.6–20.0)	11 (3–46)	15 (2–45)	58 (32–73)	86 (57–95)	97 (69–100)
**GTV-SUV40%**	1.7 (0.4–10.2)	7 (2–24)	9 (1–21)	36 (25–57)	70 (44–88)	97 (91–100)
**GTV-SUV50%**	1.2 (0.3–4.2)	4 (1–9)	6 (1–8)	25 (11–42)	60 (43–68)	97 (91–100)
**Prostate specimen**	29.1 (20.4–41.8)					
**Prostate whole**	52.3 (33.4–65.7)					

**Table 3:** DSC of different manual contouring techniques.

0–5 Team 1/Team 2	0–5 Team 1/Team 3	0–5 Team 1/Team 1v2	0–5 Team 2/Team 1v2	0–5 Team 3/Team 1v2
87.5 (81–90)	91.0 (80–98)	89.5 (78–97)	91.5 (81–97)	94.0 (80–99)
**0–10 Team1/Team 2**	**0–10 Team1/Team 3**	**0–10 Team1/Team 1v2**	**0–10 Team 2/Team 1v2**	**0–10 Team 3/Team 1v2**
88.5 (79–97)	86.5 (79–99)	88.5 (80–96)	92.0 (86–98)	90.5 (88–97)

Median and IQR are shown.

Median intraprostatic SUVmax was 39.6 (range 11.6–59.8). Analysis of semi-automatic segmentation approaches provided median volumes for GTV-SUV20–50% of 3.9, 2.6, 1.7, and 1.2 ml, respectively (**Figure 2**). GTV-SUV20% showed no significant difference to GTV-Histo and a median sensitivity of 93% and median specificity of 96% (**Figure 3**). For proportion of prostate specimen, proportion of whole prostate and coverage of GTV-Histo for semi-automatic approaches please see **Table 2**. GTV-SUV20% as best performing semi-automatic contouring approach was chosen for comparison between manual and semi-automatic contouring.

Sensitivity of GTV-SUV20% was slightly, but significantly lower than manual contouring with scaling SUVmin-max 0–5 and not significantly different to scaling SUVmin-max 0–10 and individual scaling. Specificity of GTV-SUV20% was significantly higher than GTV-Team 1 with scaling SUVmin-max 0–5 and not significantly different to other manual contouring approaches. See **Table S1** for details about p-values.

Coverage of GTV Histo was significantly higher for manual scaling SUVmin-max 0–5 than for semi-automatic contouring with SUV20%max (p < 0.024) and for scaling SUVmin-max 0–10 (p < 0.038) except for team 1. There was no significant difference between manual scaling SUVmin-max 0–10 and semiautomatic contouring.

## Discussion

Improvements in PCa detection and contouring are requested to facilitate successful biopsy guidance and focal therapy planning. PSMA-PET/CT has been established as a promising diagnostic method for identification of intraprostatic lesions (24). [^68^Ga]PSMA is a widely used tracer with excellent performance (9, 11), but new tracers like [^18^F]PSMA-1007 have been developed in recent years with putative benefits in terms of lesser renal elimination and consequent less background signal in the bladder (15), simplified manufacturing (18), and lesion detection (16). So far, there is no consensus and no recommendations on how to accurately contour intraprostatic tumor mass based on [^18^F]PSMA-1007 PET. Following the same approach as previously conducted by our group for [^68^Ga]-PSMA, this study aimed to validate different contouring methods using whole mount histology as the reference standard to be used for focal therapy planning (high sensitivity) and biopsy guidance (high specificity). Likewise, we used a quadrant-based slice-by-slice analysis approach. We chose this analysis method anticipating the most accurate analysis method but still taking mismatch susceptibilities during the registration workflow into account, which would severely effect voxel-based analysis approaches. Considering advantages of mpMRI for prostate delineation, respective contours were based on available mpMRI information (25).

Previous experience with thresholds of 30% (21), 40% (26), and 50% (27) for semi-automatic PCa segmentation and a threshold of 20% showed good performance in [^68^Ga]-PSMA/PET (11). Consequently, these approaches were selected for validation in our study. Additionally, a previously described semi-automatic segmentation techniques using a ratio between tumor and normal tissue uptake (11, 28) was utilized at the beginning, but rejected for further analysis since the volumes filled out high percentages of the prostate and performance was expected to be low.

Manually contoured GTVs with PET image scaling SUVmin-max 0–5 were statistically significantly larger than GTV-Histo (>60% larger). Therefore, we performed a second analysis, to define an additional scaling range for manual contouring, which results in volumes more concordant with GTV-Histo. The median for SUVmax was 10 and expressed in percentage relative to SUVmax median applied SUVmax was 36%. The relatively wide range of 15% to 60% in our cohort suggest, that a general recommendable threshold for threshold-based segmentation approaches may be difficult to define. However, the applied semi-automatic approaches represent the resulted SUV-range. Anticipating a putative benefit for manual contouring approaches, which does not leave out lesions below an applied threshold we consequently performed an additional analysis with manual contouring with scaling SUVmin-max 0–10.

Interobserver agreement between all Teams, using the same SUV scaling was excellent for both scaling techniques (median DSC between 0.87 and 0.94) and undermines, that using the same scaling range leads to comparable results even for readers with different levels of experience. These results comply with previous studies (11) and are contrary to MRI, the current standard of care in prostate cancer imaging, due to challenges in interpretation of different MRI modalities (23, 29). A low interobserver agreement is a prerequisite for implementing [^18^F]PSMA-1007 based tumor contours in focal therapy guidance (14, 30) or for non-invasive tumor characterization (19).

Our results of manual contouring performance reveal that volumes derived from scaling with SUVmin-max 0–10 are more consistent with GTV-Histo and sill reached high sensitivities and very high specificities, without overestimating tumor volume. GTV-SUV20% was the best performing semi-automatic contouring approach with comparable results. Nevertheless, manual scaling SUVmin-max 0–10 performed in all but one patient (patient 7) similar or better than GTV-SUV20%. Analysis of patient characteristics revealed no special features. However, intraprostatic SUVmax of this patient was 11.6, thereby lower than others and close to the applied SUVmax for scaling. This results in discrepancy of volumes in all Teams (1.6–4.8 ml), a low DSC (0.36–0.61) and plausibly in low performance of manual contouring. Regarding this aspect semi-automatic contouring possesses the advantage of easy feasibility and reproducibility. In the setting of focal therapy high sensitivities are necessary, since it’s not clear which regions represent the dominant intraprostatic lesions responsible for relapse (30, 31). On the other hand, large boost volumes and inclusion of normal tissue (low specificity) lead to an increase of toxicity. Taking these aspects into account manual contouring with scaling SUVmin-max 0–10 and semi-automatic contouring with 20% of SUVmax should be considered firstly for [^18^F]PSMA-1007-based dose escalation. In case intraprostatic SUVmax is close to the applied SUVmax for manual scaling, adjustment of the range, for instance SUVmin-max 0–5, might be considered as an appropriate alternative. Our results suggest, that the use [^18^F]PSMA-1007 for contouring of lesions for focal therapy planning is likely to be as effective as other tracers, who’s performance was evaluated in radiotherapy planning studies and showed promising results in terms of tumor control and normal tissue toxicities (14, 32).

Another requirement for sufficient tumor control is coverage of intraprostatic tumor. Manual contouring and GTV-SUV20% reached a median coverage of GTV-Histo of >50% in all patients. Coverage with scaling SUVmin-max 0–5 was significantly higher, again explainable due to the large and overestimated volumes. Comparison of remaining approaches showed no significant difference. However, the co-registration workflow bears uncertainties in the exact localization of GTV-Histo. Consequently, coverage of GTV-Histo calculated by intersection volumes is the most inaccurate parameter of this study and conclusions based on the coverage of GTV-Histo should be drawn with caution. Nevertheless, the recommended contouring approaches reveal volumes consistent with GTV-Histo. Considering the fact that PSMA-Expression shows heterogeneity with potentially low or even missing PSMA-expression (33, 34), information provided by mpMRI complements PSMA-PET for intraprostatic tumor detection (11). As previously demonstrated combination of mpMRI and PSMA-PET/CT further achieves higher sensitivity and specificity (11, 12, 35, 36). Consequently. future studies should investigate whether the addition of [^18^F]PSMA-1007 information for focal therapy planning can be translated into increased tumor control.

Biopsy guidance in patients with PCa relies on high specificities, which increases the chance of PCa detection in the biopsy sample. As expected, specificity of manual scaling SUVmin-max 0–10 and SUVmax20% was statistically significantly higher than scaling with SUVmin-max 0–5. Volumes of GTV-50%, but not GTV-SUV20–40% were significantly smaller than GTV-Histo, however higher thresholds yielded to less coverage of GTV-Histo. Bravaccini et al. showed that PSMA-Expression correlates with Gleason Score (37), therefore targeting lesions with high SUV values might guide to more aggressive PCa regions. Semi-automatic scaling approach with 30–40% of SUVmax showed good sensitivity with excellent specificity and might be effective and feasible to target lesions that are more aggressive. Consequently, semiautomatic contouring with 30–40% of SUVmax are recommended for [^18^F]PSMA-1007-PET guided biopsies, depending on the obtained volumes. However, scaling with SUVminmax 0–10 might be an appropriate alternative.

This study shows a trend towards higher sensitivity and specificity in intraprostatic PCa detection for [^18^F]PSMA-1007 compared to [^68^Ga]-PSMA. Histopathological comparison studies showed sensitivities between 64 and 89% and specificities between 71 and 95% for [^68^Ga]-PSMA (11, 12, 36, 38, 39). Our results performed similarly well as a study by Kuten et al., which showed a sensitivity of 100% and a specificity of 90.9% for [^18^F]PSMA-1007. In the head-to-head comparison [^18^F]PSMA-1007 showed a higher sensitivity and lower specificity than [^68^Ga]-PSMA (85.7 and 98.2%, respectively). Noteworthy, both tracers detected significant index lesions equally. However, identification of PCa was based on expert review with unknown scaling (16), which hampers direct comparison to our study. Kesch et al. showed lower sensitivity (71%) and specificity (81%) for [^18^F]PSMA-1007 (35). A possible explanation for these variations might be the usage of different approaches for coregistration and analysis. Whether these aspects contribute significantly to the results has not been challenged yet. Future studies should compare these to clarify comparability. Furthermore future studies should evaluate performance of neuronal networks trained for GTV contouring in [^18^F]PSMA-1007 images, which might circumvent the issue of proper manual scaling ranges.

The study’s limitation is the imprecision in correlation of PET/CT and histopathology (e.g. non-linear shrinkage of the prostate after prostatectomy). As mentioned, low to moderate coverage of GTV-Histo might be a consequence of mismatch in coregistration or incomplete histopathological coverage. This potential bias is marginal for calculation of sensitivities and specificities, since they were not performed on a voxel-level but on a less stringent slice-by-slice level. Furthermore, the use of two different PET/CT scanners is a limitation. However, a phantom study confirmed the comparability of SUV values between the two scanning systems and rigorous reconstruction parameters were applied. Additionally, 9 of 10 patients underwent the scan in the same scanner and the single outlier patient was independent of the used scanner type. Third, the sample size in our study is relatively small, due to the elaborate pathology-imaging co-registration protocol. Lastly, we enrolled patients planned for prostatectomy to obtain histopathologic information from the specimens and thus caused a selection bias. Consequently, our results are only representative for intermediate- and high-risk PCa patients. However, these patients are likely to benefit most from focal therapy approaches, which is being investigated in phase III trials (4).

In conclusion manual contouring by using the same PET image scaling technique yields low interobserver variability even for readers with different levels of experience. Scaling PET images with SUVmin-max:0–5 showed excellent sensitivities but moderate specificity by overestimating the tumor volume. PET image scaling with SUVmin-max 0–10 showed slightly but statistically significant lower sensitivities with statistically significant higher specificities. Semi-automatic contouring with SUVmax20% similarly achieved high sensitivity and very high specificity. In this study manual scaling with SUvmin-max 0–10 performed similar or better than SUVmax20% in all but one patient. However, semiautomatic contouring approaches possess the advantage of easy feasibility and reproducibility. Consequently, evaluating total performance manual contouring with SUVmin-max 0–10 and semiautomatic contouring applying a threshold of 20% of SUVmax are firstly recommended for [^18^F]PSMA-1007 based focal therapy approaches. Providing very high specificities and depiction of the high-uptake areas within the tumor, semi-automatic approaches applying thresholds of SUVmax 30–40% are recommend for biopsy guidance.

## Data Availability Statement

The raw data supporting the conclusions of this article will be made available by the authors upon request without undue reservation.

## Ethics Statement

The studies involving human participants were reviewed and approved by Institutional Review Board University Freiburg. The patients/participants provided their written informed consent to participate in this study.

## Author Contributions

SS and CZ contributed to conception and design of the study. MK and AS enrolled patients. MK and CZ performed histo/image co-registration. SS, MK, LC, CZ, and AG performed PET-contouring. SS and MK conducted metrics calculation. WS-S, CJ, and AS were responsible for surgery indication and prostatectomy. SK and PB conducted the histopathological processing and marking of tumor lesions. TF and JR were responsible for conduction and reporting of the PSMA-PET/CTs. TS, NN, and AG supervised the study and co-registration. SS wrote the first draft of the manuscript. CZ wrote sections of the manuscript. All authors contributed to the article and approved the submitted version.

## Funding

This study was funded by the Era PerMed call 2018 (BMBF).

## Conflict of Interest

The authors declare that the research was conducted in the absence of any commercial or financial relationships that could be construed as a potential conflict of interest.

The reviewer TZ declared a past co-authorship with several of the authors WS-S, CJ, and AG to the handling editor.
