# Supplementary material for: Comparison of Manual and Semi-Automatic [18F]PSMA-1007 PET Based Contouring Techniques for Intraprostatic Tumor Delineation in Patients With Primary Prostate Cancer and Validation With Histopathology as Standard of Reference
Source: Front Oncol. 2020 Dec 7;10:600690. doi: 10.3389/fonc.2020.600690 (PMC7750498; doi:10.3389/fonc.2020.600690)
Supplement: Supplementary file 1 [file Table_1.docx]

| **Sensitivity** | | | | | | | | | | |
| --- | --- | --- | --- | --- | --- | --- | --- | --- | --- | --- |
|  | **Team1 0-5** | **Team2 0-5** | **Team3 0-5** | **Team 1v2 0-5** | **Team1 0-10** | **Team2 0-10** | **Team 3 0-10** | **Team 1v2 0-10** | **Indivi-dual** | **SUV20%** |
| **Team1 0-5** |  | 0.767 | 0.605 | 0.684 | **0.024** | **0.001** | **0.007** | **0.009** | **0.001** | **0.003** |
| **Team2 0-5** |  |  | 0.824 | 0.912 | 0.050 | **0.002** | **0.016** | **0.020** | **0.003** | **0.007** |
| **Team3 0-5** |  |  |  | 0.912 | 0.083 | **0.004** | **0.029** | **0.035** | **0.006** | **0.013** |
| **Team 4 0-5** |  |  |  |  | 0.064 | **0.003** | **0.022** | **0.027** | **0.005** | **0.010** |
| **Team1 0-10** |  |  |  |  |  | 0.237 | 0.657 | 0.711 | 0.318 | 0.460 |
| **Team2 0-10** |  |  |  |  |  |  | 0.460 | 0.420 | 0.854 | 0.658 |
| **Team 3 0-10** |  |  |  |  |  |  |  | 0.941 | 0.580 | 0.767 |
| **Team 4 0-10** |  |  |  |  |  |  |  |  | 0.530 | 0.712 |
| **Individual** |  |  |  |  |  |  |  |  |  | 0.800 |
| **Specificity** | | | | | | | | | | |
|  | **Team1 0-5** | **Team2 0-5** | **Team3 0-5** | **Team 1v2 0-5** | **Team1 0-10** | **Team2 0-10** | **Team 3 0-10** | **Team 1v2 0-10** | **Indivi-dual** | **SUV20%** |
| **Team1 0-5** |  | 0.907 | 0.371 | 0.640 | **0.000** | **0.003** | **0.005** | **0.002** | **0.006** | **0.043** |
| **Team2 0-5** |  |  | 0.436 | 0.726 | **0.005** | **0.005** | **0.006** | **0.003** | **0.008** | 0.057 |
| **Team3 0-5** |  |  |  | 0.669 | **0.006** | **0.006** | 0.052 | **0.027** | 0.062 | 0.259 |
| **Team 4 0-5** |  |  |  |  | **0.002** | **0.002** | **0.018** | **0.008** | **0.021** | 0.120 |
| **Team1 0-10** |  |  |  |  |  | >0.999 | 0.436 | 0.613 | 0.392 | 0.111 |
| **Team2 0-10** |  |  |  |  |  |  | 0.436 | 0.613 | 0.392 | 0.111 |
| **Team 3 0-10** |  |  |  |  |  |  |  | 0.785 | 0.938 | 0.414 |
| **Team 4 0-10** |  |  |  |  |  |  |  |  | 0.726 | 0.276 |
| **Individual** |  |  |  |  |  |  |  |  |  | 0.460 |

Supplementary Material

# Supplementary Table S1

Crosstable shows p-values for sensitivity and specificity comparing manual contouring approaches and GTV-SUV20%. Bold indicates significance (p<0.05).
